# Night-Warming Priming at the Vegetative Stage Alleviates Damage to the Flag Leaf Caused by Post-anthesis Warming in Winter Wheat (*Triticum aestivum* L.)

**DOI:** 10.3389/fpls.2021.706567

**Published:** 2021-10-06

**Authors:** Yonghui Fan, Zhaoyan Lv, Ting Ge, Yuxing Li, Wei Yang, Wenjing Zhang, Shangyu Ma, Tingbo Dai, Zhenglai Huang

**Affiliations:** ^1^College of Agronomy, Anhui Agricultural University/Key Laboratory of Wheat Biology and Genetic Improvement on South Yellow and Huai River Valley, Ministry of Agriculture, Hefei, China; ^2^College of Horticulture, Anhui Agricultural University, Hefei, China; ^3^Key Laboratory of Crop Physiology, Ecology and Production Management, Nanjing Agricultural University, Nanjing, China

**Keywords:** grain-filling stage, night warming, wheat, senescence, yield

## Abstract

The asymmetric warming in diurnal and seasonal temperature patterns plays an important role in crop distribution and productivity. Asymmetric warming during the early growth periods of winter wheat profoundly affects its vegetative growth and post-anthesis grain productivity. Field experiments were conducted on winter wheat to explore the impact of night warming treatment in winter (Winter warming treatment, WT) or spring (Spring warming treatment, ST) on the senescence of flag leaves and yield of wheat plants later treated with night warming during grain filling (Warming treatment during grain filling, FT). The results showed that FT decreased wheat yield by reducing the number of grains per panicle and per 1,000-grain weight and that the yield of wheat plants treated with FT declined to a greater extent than that of wheat plants treated with WT + FT or ST + FT. The net photosynthetic rate, chlorophyll content, and chlorophyll fluorescence parameters of the flag leaves of wheat plants treated with WT + FT or ST + FT were higher than those under the control treatment from 0 to 7 days after anthesis (DAA) but were lower than those under the control treatment and higher than those of wheat plants treated with FT alone from 14 to 28 DAA. The soluble protein and Rubisco contents in the flag leaves of wheat plants treated with WT + FT or ST + FT were high in the early grain-filling period and then gradually decreased to below those of the control treatment. These contents were greater in wheat plants treated with WT + FT than in wheat plants treated with ST + FT from 0 to 14 DAA, whereas the opposite was true from 21 to 28 DAA. Furthermore, WT + FT and ST + FT inhibited membrane lipid peroxidation by increasing superoxide dismutase and peroxidase activities and lowering phospholipase D (PLD), phosphatidic acid (PA), lipoxygenase (LOX), and free fatty acid levels in the early grain-filling period, but their inhibitory effects on membrane lipid peroxidation gradually weakened during the late grain-filling period. Night-warming priming alleviated the adverse effect of post-anthesis warming on yield by delaying the post-anthesis senescence of flag leaves.

## Introduction

In the twentieth century, the annual mean temperature in China increased from 0.4 to 0.8°C, and the mean temperature increases in spring, summer, fall, and winter were 0.33, 0.40, 0.73, and 1.37°C, respectively. China experienced 17 consecutive warm winters beginning in 1990, and the mean temperature increase in winter was more than two times the average annual increase in global temperature (0.6°C) (Qin et al., 2010; Gao et al., 2011; Hatfield et al., 2011). According to the prediction by the Intergovernmental Panel on Climate Change (Haines, 2003), climate warming in China will continue in the twenty-first century, especially in the winter half-year in northern China. Increasingly drastic climate change will greatly affect atmospheric circulation and cause more extreme weather events (Zhao et al., 2010; You et al., 2011). It is expected that these extreme weather events will occur to a greater extent and at higher frequencies in East China because the warming rate in this region is higher than the global average (Ren et al., 2008).

Wheat is one of the three major crops in the world and the leading food crop in China, and its yield plays a key role in the food security and socioeconomic development of China (Slafer, 2003). High temperature in the post-anthesis period often accelerates wheat maturation and, thus, severely reduces wheat yield and quality (Riaz et al., 2021). Grain number is mainly determined during the period immediately preceding anthesis, and individual grain weight is defined during the grain-filling period (Karim et al., 2020). Temperature is an important environmental factor affecting grain filling during grain formation (Hütsch et al., 2018; Osman et al., 2020). Shirdelmoghanloo et al. (2016) found that warming in the early post-anthesis period significantly reduces photosynthesis and the antioxidant capacity of flag leaves in wheat, resulting in smaller grain size and weight but barely affecting grain yield. Bian et al. (2012) found that a temperature increase of approximately 1.8°C after anthesis, with an average daily temperature of approximately 20.8°C and an average daytime temperature of approximately 23.1°C, was conducive to protein accumulation, with the highest increase coming in grain albumins, while a temperature increase of <1.8°C barely affected the starch content. Huebner and Bietz (1988) reported that, during wheat grain filling, the most suitable ambient temperature is 20–24°C. An ambient temperature higher than 25°C shortens the grain-filling period, thus affecting grain weight, while an ambient temperature lower than 12°C causes chilling injuries in wheat.

Crop yield is closely related to the net photosynthetic assimilation process during the grain-filling period in wheat. The photosynthesis of flag leaves contributes the most to the final grain dry weight in winter wheat. Hence, the study of flag leaves in response to climate warming occurring during the winter and spring seasons is crucial. The main source of mass accumulation in wheat grain is the photosynthetic products of flag leaves during the grain-filling period, and the physiological activities of these leaves directly affect dry matter accumulation and transport (Feng et al., 2014). Grain filling is also affected by the photosynthetic characteristics of flag leaves, which, in turn, affects grain weight and yield (Prieto et al., 2009). The soluble protein content in plant leaves reflected the nitrogen content, and the nitrogen content in leaves had a positive relation with photosynthetic capacity. Most of the nitrogen in plants is stored in enzymes participating in photosynthesis, especially in Rubisco, which is a major source of nitrogen recycling (Makino et al., 1997; Masclaux-Daubresse et al., 2008). However, in the middle and late grain-filling periods of wheat, flag leaf senescence often occurs. During the grain-filling stage, programmed leaf senescence occurs, accompanied by a burst of excessive reactive oxygen species (ROS), such as superoxide and hydroxyl radicals (Kong et al., 2015, 2017). The significant declines in chlorophyll content (Lee et al., 2001), protein content, and the activities of various antioxidant enzymes in wheat during senescence break the dynamic balance between the generation and elimination of ROS in plants, causing the accumulation of large amounts of ROS, damage to biological macromolecules, and the destruction of membrane lipid structures, thus hindering the normal operation of photosynthesis (Huffaker, 1990).

A high temperature in the late growth stage reduces the photosynthetic capacity of wheat leaves, intensifies membrane lipid peroxidation, accelerates plant senescence, and leads to lower wheat yield (Savin and Nicolas, 1996). Suitable stress stimuli during the early reproductive period can increase the resistance of crops to the same or other stresses (Bruce et al., 2007). Li et al. (2011) reported that a pre-anthesis waterlogging pretreatment improved the resistance of wheat plants to post-anthesis waterlogging, thereby significantly increasing yield. Another study exposed wheat seedlings at 21 days after sowing to moderate drought for 9 days and then allowed them to recover for 1 day. These drought-adapted seedlings showed a stronger drought resistance than non-drought-adapted seedlings when they were later exposed to severe drought stress for 9 days (Selote and Khanna-Chopra, 2006).

There is a lack of relevant research on whether warming in the early reproductive period can improve the viability of wheat in the late reproductive period. In the present study, field experiments were conducted to investigate the impact of a moderate night-warming treatment in winter (WT) or spring (ST) on the senescence of flag leaves and the yield of wheat plants that were later treated with night warming during the grain-filling period (FT). The mechanism underlying these effects was investigated by determining the photosynthetic and oxidative metabolism characteristics of flag leaves and their yield output.

## Materials and Methods

### Experimental Design

The field experiments were conducted at the Jiangpu Experimental Station of Nanjing Agricultural University from 2013 to 2014. Two local cultivars, vernal type Yangmai 13 (YM13) and facultative type Yannong 19 (YN19), were used. The soil in the test plots before sowing contained 21.62 g kg^−1^ of organic matter, 1.12 g kg^−1^ of total nitrogen, 14.39 mg kg^−1^ of available nitrogen, 17.4 mg kg^−1^ of available phosphorus, and 115.52 mg kg^−1^ of available potassium. The plot size was 8 m^2^ (2 m × 4 m) with a row spacing of 25 cm. The randomized block design was adopted for field experiments with three replications. A total of 240 kg ha^−1^ of nitrogen was applied in three applications over the whole growth period. The basal: topdressing ratio (basal fertilizer:jointing fertilizer:booting fertilizer) was 5:3:2. Superphosphate (P_2_O_5_) and potassium oxide (K_2_O) fertilizers were applied together as the basal fertilizers at 100 and 150 kg ha^−1^, respectively, during the wheat growth period. The seeds were sown on October 26, 2013. The seedlings were then thinned to a density of 180 m^−2^ when they sprouted three leaves. Plants were carefully noted to have reached a particular growth stage when more than 50% of the wheat plants reached that stage (tillering: Zadoks growth stage 21, main shoot and one tiller; jointing: Zadoks growth stage 31, the first node was detectable; booting: Zadoks growth stage 41, flag leaf sheath-extending stage; anthesis: Zadoks growth stage 60, the beginning of pollination). The stages were determined according to Zadoks et al. (1974). The phenophases of the two wheat cultivars are provided in Table 1. The days taken from flowering to grain formation were recorded when more than 50% of the plants completed their flowering to the physiological maturity of the crop. Uniform tillers flowering on the same day were tagged for sampling and measurements. The rest of the management measures were the same as those used for the high-yield cultivation of field crops.

**Table 1:** Phenophase of the two wheat cultivars.

**Cultivar**	**Phenophase date (day/month)**				
	**Sowing**	**Stem-elongation**	**Booting**	**Anthesis**	**Maturity**
Yangmai 18	26th October 2013	21st February 2014	22nd March 2014	13rd April 2014	20th May 2014
Yannong 19	26th October 2013	28th February 2014	28th March 2014	19th April 2014	24th May 2014

**Figure 1:**
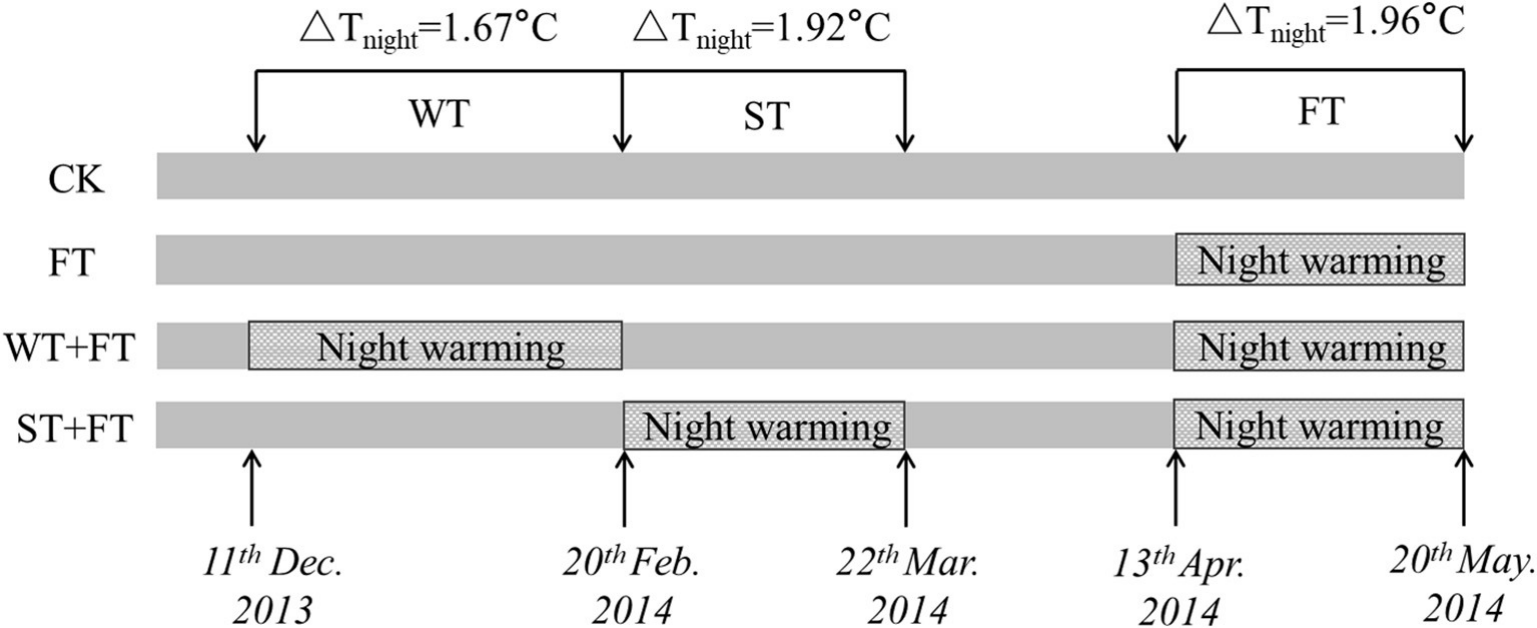
Schematic of the setup of different warming treatments. CK: control; FT: night warming treatment during the grain-filling period; WT + FT: night warming treatment during winter and the grain-filling period; ST + FT: night warming treatment during spring and the grain-filling period. NW: night warming. ΔT_night_ refers to the increase of mean night temperature between treatments and the control. Mean night temperature is the mean of all temperature data on a 10 min interval from 19:00 p.m. to 07:00 a.m.

**Figure 2:**
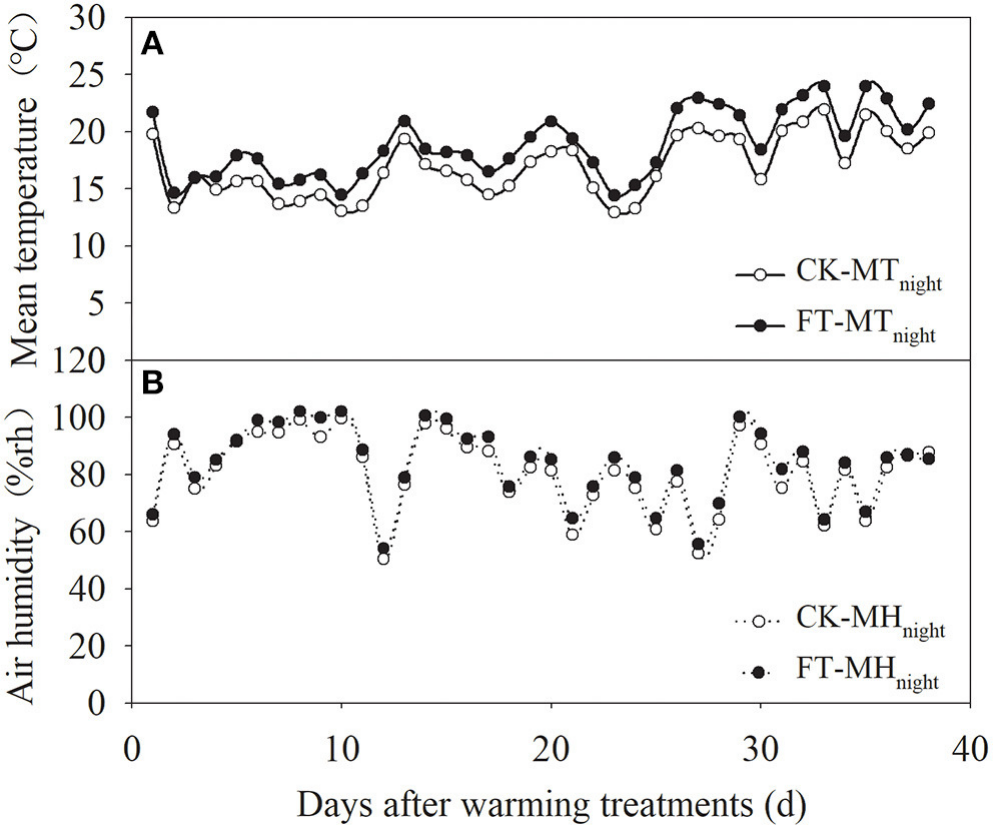
Mean nighttime canopy temperature **(A)** and air humidity **(B)** under FT (anthesis period-maturation period).

Passive night warming (Shaver et al., 1998; Beier et al., 2004; DeMarco et al., 2014; Deslippe et al., 2015) was adopted in this study using a warming facility (length: 3 m, width: 5 m, and height: 2 m) with a removable plastic membrane. Designated personnel rolled down the plastic membrane at sunset to cover the warmed plots and rolled it up at sunrise to expose the warmed plots to the natural environment (from around 19:00 p.m. to 07:00 a.m. of the next day). The facility was equipped with a ventilation system to ensure the normal respiration and ventilation of the crops at night. To ensure that each plot received the same precipitation, the plastic membrane was rolled up at night when it rained or snowed. A dual-channel thermometer (NZ-LBR-F, Nanjing Nengzhao Electronic Instrument Co., Ltd., China) was used to record the canopy night temperature of warmed plots once every 10 min during the wheat growth period. Figure 1 shows the setup of the different warming treatments. The four treatments included non-warming (CK), night-warming treatment during the grain-filling period only (FT: anthesis to maturity), night-warming treatment during winter (WT: tillering to jointing) and the grain-filling period (WT + FT), and night-warming treatment during spring (ST: jointing to booting) and the grain-filling period (ST + FT). The mean nighttime temperatures of the winter wheat canopy increased by 1.67 (WT) and 1.92°C (ST), respectively. FT increased the mean nighttime temperature of the winter wheat canopy by 1.96°C. The temperature and humidity data during the warming period are shown in Figure 2.

### Measures and Methods

#### Yield and Yield Components

Two plants of 2 m long rows (1 m^2^) were marked at anthesis in the center of the plots to measure grain yield at maturity. Spike number per m^2^ was counted at physiological maturity, and the plants were cut with a sickle at the soil level. Harvested plants were carefully threshed, dried, and weighed to measure grain yield in kg ha^−1^ at 14% moisture. A total of 50 culms from the four rows in the middle of each plot were sampled at the soil level to measure grain number, grain weight per spike, and 1,000-grain weight.

#### The Net Photosynthetic Rate (Pn)

The net photosynthetic rate of flag leaves was measured at 0, 7, 14, 21, 28, and 35 days after anthesis (DAA). Four flag leaves with uniform growth and similar light-incidence directions were measured for each treatment with three replications. The *Pn* of wheat leaves was measured using an LI-6400 portable photosynthesis system (Li-Cor Inc., Lincoln, NE, USA). The chamber had an opening for air and a red/blue light source. The photosynthetically active radiation (PAR) was set to 1,200 μmol m^−2^ s^−1^, and the CO_2_ concentration was approximately 380 μmol L^−1^.

#### Chlorophyll Content

One gram of leaves was cut into several sections and placed in 50 ml of acetone:ethanol (v:v = 1:1) extracting solution in the dark at 25°C for 24 h, and the optical density (OD) of the extracts was measured at 470, 663, and 645 nm. The chlorophyll content was calculated according to the formula of Zheng et al. (2009).

#### Chlorophyll Fluorescence Kinetics Parameters

The chlorophyll fluorescence kinetics parameters of the same flag leaves used in the photosynthesis measurements were measured with a chlorophyll fluorometer (PAM-2500, Heinz Walz GmbH, Effeltrich, Germany). The minimum and maximum chlorophyll fluorescence (F_0_ and F_m_, respectively) were measured after dark adaptation for 30 min. Steady-state fluorescence (F_s_) was measured after irradiation with 1,200 μmol m^−2^ s^−1^ for 10 min and a strong flash. The maximum fluorescence under light adaptation (F_m_′) was recorded. After dark adaptation for 3 s, the far-red light was turned on, and the initial fluorescence F_0_′ under light adaptation was measured to calculate the effective quantum yield of photosystem II (PSII) photochemistry (Φ_PSII_). Parameters were calculated referring to the maximum efficiency of PSII photochemistry under dark-adapted (F_v_/F_m_), F_v_/F_m_ = (F_m_-F_0_)/F_m_, where F_v_ is the variable chlorophyll fluorescence and Φ_PSII_ = (F_m_′-F_0_′)/F_m_′ (Genty et al., 1989).

#### Soluble Protein and Rubisco Content

Soluble protein content measurement followed the procedure of Lowry et al. (1951) with little change. Frozen samples (0.5 g) were extracted in a sodium phosphate buffer (50 mM, pH 7). The extracts were centrifuged (4,000 × *g*, 10 min, and 4°C). Soluble proteins were quantified using the supernatants of the samples, with bovine serum albumin as a standard. Rubisco was analyzed using sodium dodecyl sulfate-polyacrylamide gel electrophoresis (SDS-PAGE) as described previously (Makino et al., 1986), with few changes. Frozen tissue was homogenized in 50 mM of a Tris–HCl buffer (pH 8) containing 5 mM of β-mercaptoethanol and 12.5% of (v/v) glycerol and then centrifuged at 15,000× *g* for 15 min. To the supernatant, we added SDS, β-mercaptoethanol, and glycerol to final concentrations of 1% (w/v), 2% (v/v), and 5% (v/v), respectively, and then boiled the mixture for 5 min. A sample of this preparation was used for electrophoresis. For protein visualization, the gels were stained with 0.1% (w/v) Coomassie Brilliant Blue R-250. The stained bands were excised from the gels with a razor blade and eluted in 1 ml of formamide in a stoppered amber test tube at 50°C for 8 h with no stained gel as a standard. The absorbance of the resultant solution was read at 595 nm with a spectrophotometer (UV-4806S, Unico Instrument, Shanghai, China). This part has been revised accordingly.

#### Antioxidant Capacity of the Leaves

The rate of superoxide anion radical (${\text{O}}_{2}^{-}$) production was measured according to Sui et al. (2007). Superoxide dismutase (SOD, EC 1.15.1.1) activity was measured according to Yang et al. (2007), and peroxidase (POD, EC 1.11.1.7) activity was determined according to Zheng et al. (2009). Malondialdehyde (MDA) content was measured according to Zheng et al. (2009). The above measurements were carried out in three biological replicates (leaves).

#### Membrane Phospholipid Metabolism

The phospholipase D, PA, LOX, and FFA concentrations were determined using the double-antibody sandwich ELISA using the reagent kit DRE97194 (Fengxiang Biotech, Shanghai, China).

### Data Processing

All data are expressed as the means of three replicates. A one-way ANOVA was performed for the PLD, PA, LOX, and FFA contents to assess the significant differences between temperature treatments. A two-way ANOVA was performed on grain yield and its components, *Pn*, chlorophyll content, Fv/Fm, Φ_PSII_, soluble protein content, Rubisco content, ${\text{O}}_{2}^{-}$ production rate, malondialdehyde (MDA) content, SOD activity, and POD activity to identify the significant differences between the cultivars and temperature treatments. Statistical analyses were conducted using the Statistical Package for the Social Sciences (SPSS) software (SPSS ver. 10, SPSS, Chicago, IL, USA). The results were plotted in SigmaPlot 10.0.

## Results

### Yield and Yield Components

Table 2 shows that the three warming treatments reduced the yields of the two wheat cultivars, and in terms of yield, the treatments followed the descending order of CK > WT + FT > ST + FT > FT, indicating that FT was not conducive to yield. In particular, the WT and ST priming treatments reduced the inhibitory effect of FT on grain yield. Compared with control, FT had a similar spike number but significantly lower grain number, 1,000-grain weight, and, therefore, yield (minus 17%). On the other hand, the spike number, grain number, and 1,000-kernel weight in primed plants were like those of the control, and yield losses, especially in the ST samples, were limited.

**Table 2:** Impact of a night warming treatment in winter (WT) or spring (ST) on the grain yield and yield components of wheat later treated with night warming treatment during the grain-filling period (FT).

**Cultivar**	**Treatment**	**Spikes (×10**^**4**^ hm^**−2**^**)**	**Grain number (spike** ^**−1**^ **)**	**1000-grain weight (g)**	**Grain yield (kg hm** ^**−2**^ **)**
YM13	CK	450.33 a	47.56 a	41.54 a	7297.80 a
	FT	446.00 a	44.86 b	38.77 c	6079.67 d
	WT+FT	428.00 a	47.81 a	40.18 b	6536.13 c
	ST+FT	438.67 a	48.19 a	40.96 ab	6790.66 b
YN19	CK	497.67 a	44.40 a	39.08 a	6832.73 a
	FT	492.00 a	41.56 b	36.64 b	5645.86 c
	WT+FT	473.33 abc	43.26 ab	37.95 ab	5789.74 c
	ST+FT	479.67 ab	43.72 a	38.34 ab	6175.65 b
*F-*value	*F-*_Cultivar_	43.013^**^	127.864^**^	69.469^**^	104.551^**^
	*F-*_Treatment_	2.247	14.935^**^	15.627^**^	86.315^**^
	*F-*_C × *T*_	0.036	1.158	0.152	1.697

*Different letters in the same column indicate the differences between treatments at p < 0.05. F-_Cultivar_ and F-_Treatment_ represent the F value of the cultivar and the warming treatment, respectively; F-_C × T_ represents the F value of the interaction between the cultivar and temperature*.

** *represent significance at the 0.01 level*.

### Pn and Chlorophyll Content

**Figure 3:**
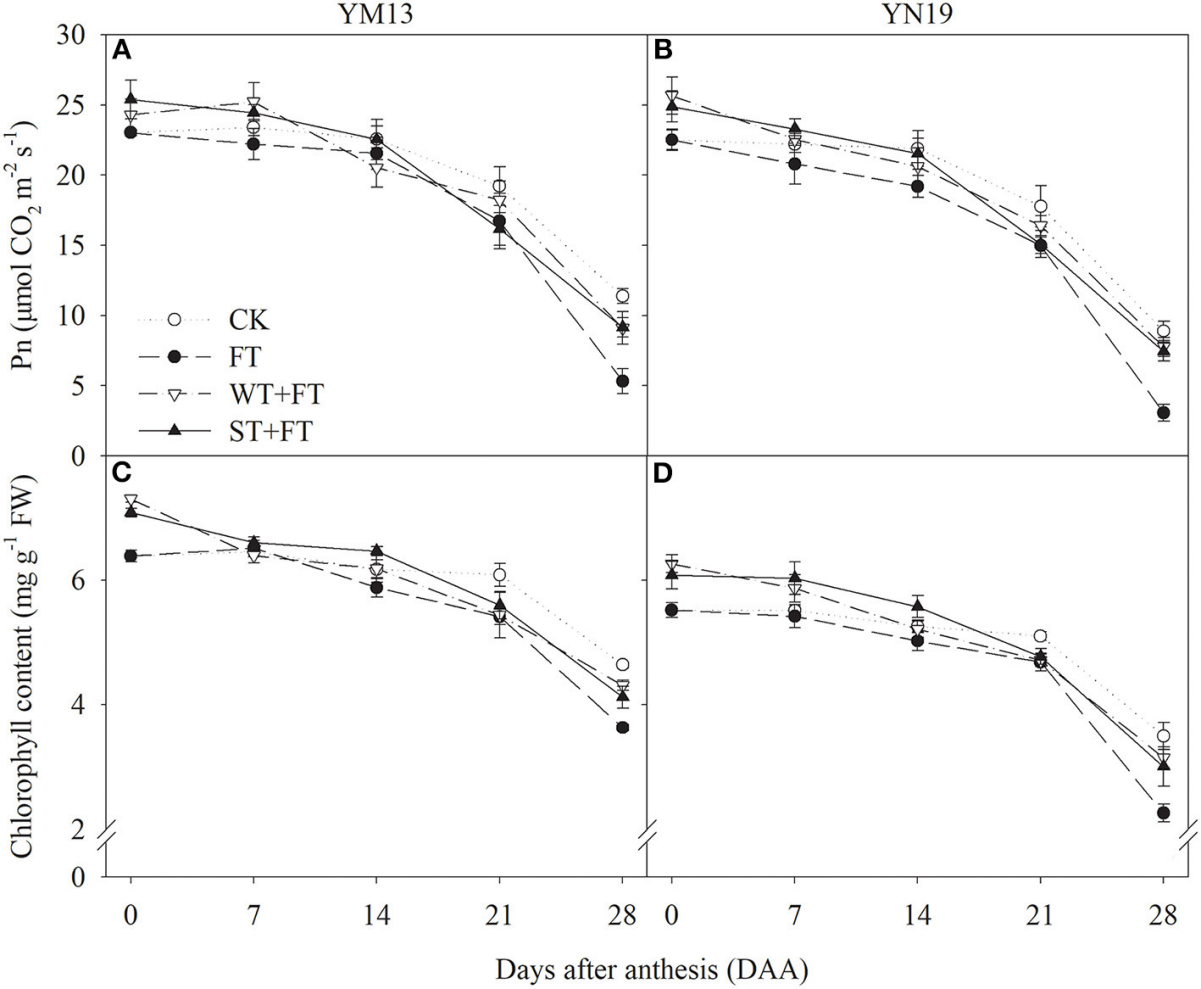
Impact of night warming treatment in winter or spring on the net photosynthetic rate (*Pn)* **(A,B)** and chlorophyll content **(C,D)** of wheat flag leaves later treated with night warming during the grain-filling period.

Figures 3A,B shows the *Pn* values of wheat flag leaves under WT + FT and ST + FT were higher than those under the control treatment from 0 to 7 DAA and were lower than those under the control treatment, but still higher than those under FT from 14 to 28 DAA. At 28 DAA, the *Pn* values of the flag leaves in primed plants were significantly higher than those under the FT of the two cultivars. There was a significant difference in the flag leaf *Pn* between warming treatments at 0–7 and 21–28 DAA (*p* < 0.01; Table 3). Figures 3C,D shows that the flag leaf chlorophyll content in the two cultivars was higher under WT + FT and ST + FT than under the control treatment from 0 to 7 DAA and was lower than the control from 21 to 28 DAA. Night-warming priming increased the post-anthesis chlorophyll content in flag leaves than that under FT. There was a significant difference in the flag leaf chlorophyll contents between warming treatments and cultivars at 0–28 DAA (*p* < 0.01; Table 3).

**Table 3:** Two-way ANOVA analysis for *Pn*, chlorophyll content, Fv/Fm, Φ_PSII_, soluble protein content, Rubisco content, ${\text{O}}_{2}^{-}$ production rate, MDA content, SOD activity, and POD activity of the two cultivars as affected by a night warming treatment and the interactive effect.

**Time**	***Pn***			**Chlorophyll content**			**F**_**v**_ **/F**_**m**_			**Φ**_**PSII**_			**Soluble protein**			**Rubisco content**			${\text{O}}_{2}^{-}$ **production rate**			**MDA content**			**SOD activity**			**POD activity**		
	**C**	**T**	**C × T**	**C**	**T**	**C × T**	**C**	**T**	**C × T**	**C**	**T**	**C × T**	**C**	**T**	**C × T**	**C**	**T**	**C × T**	**C**	**T**	**C × T**	**C**	**T**	**C × T**	**C**	**T**	**C × T**	**C**	**T**	**C × T**
0 DAA	ns	^**^	ns	^**^	^**^	ns	ns	^**^	^*^	ns	^**^	^*^	^**^	^**^	ns	^**^	^**^	ns	ns	^**^	ns	ns	^**^	^*^	ns	^**^	ns	^**^	^**^	^*^
7 DAA	^**^	^**^	ns	^**^	^**^	^*^	ns	^**^	ns	^*^	^*^	ns	^**^	^**^	ns	^**^	^**^	ns	^**^	^*^	ns	ns	^*^	ns	^*^	^*^	ns	ns	^**^	ns
14 DAA	^*^	^*^	ns	^**^	^**^	ns	^**^	^**^	^**^	^**^	^*^	ns	^*^	^**^	ns	^**^	^*^	^**^	ns	^**^	ns	ns	^*^	ns	ns	ns	ns	^**^	^**^	^**^
21 DAA	^*^	^**^	ns	^**^	^**^	ns	^**^	^**^	^*^	^**^	^**^	^*^	^**^	^**^	ns	ns	^**^	^**^	^**^	^**^	^**^	^**^	^**^	^*^	^**^	^**^	ns	^**^	^**^	ns
28 DAA	^**^	^**^	ns	^**^	^**^	ns	ns	^**^	^**^	^**^	^**^	^**^	^**^	^**^	^**^	ns	^**^	^**^	ns	^**^	ns	^**^	^**^	^**^	^**^	^**^	ns	^*^	^**^	^**^

Pn, F_v_/F_m_, Φ_PSII_, ${\text{O}}_{2}^{•-}$, MDA, SOD, and POD refer to net photosynthetic rate, maximum efficiency of photosystem II (PS II) photochemistry under dark adapted, the effective quantum yield of PS II photochemistry, superoxide anion radical, malondialdehyde, superoxide dismutase, and peroxidase, respectively. DAA refers to days after anthesis. ^*^, ^**^, and ns indicate being significant at 0. 05, 0.01 levels, and no significant difference, respectively. C, T, and C × T refer to cultivar, treatment, and the interaction of cultivar with treatment, respectively.

### Chlorophyll Fluorescence Parameters

**Figure 4:**
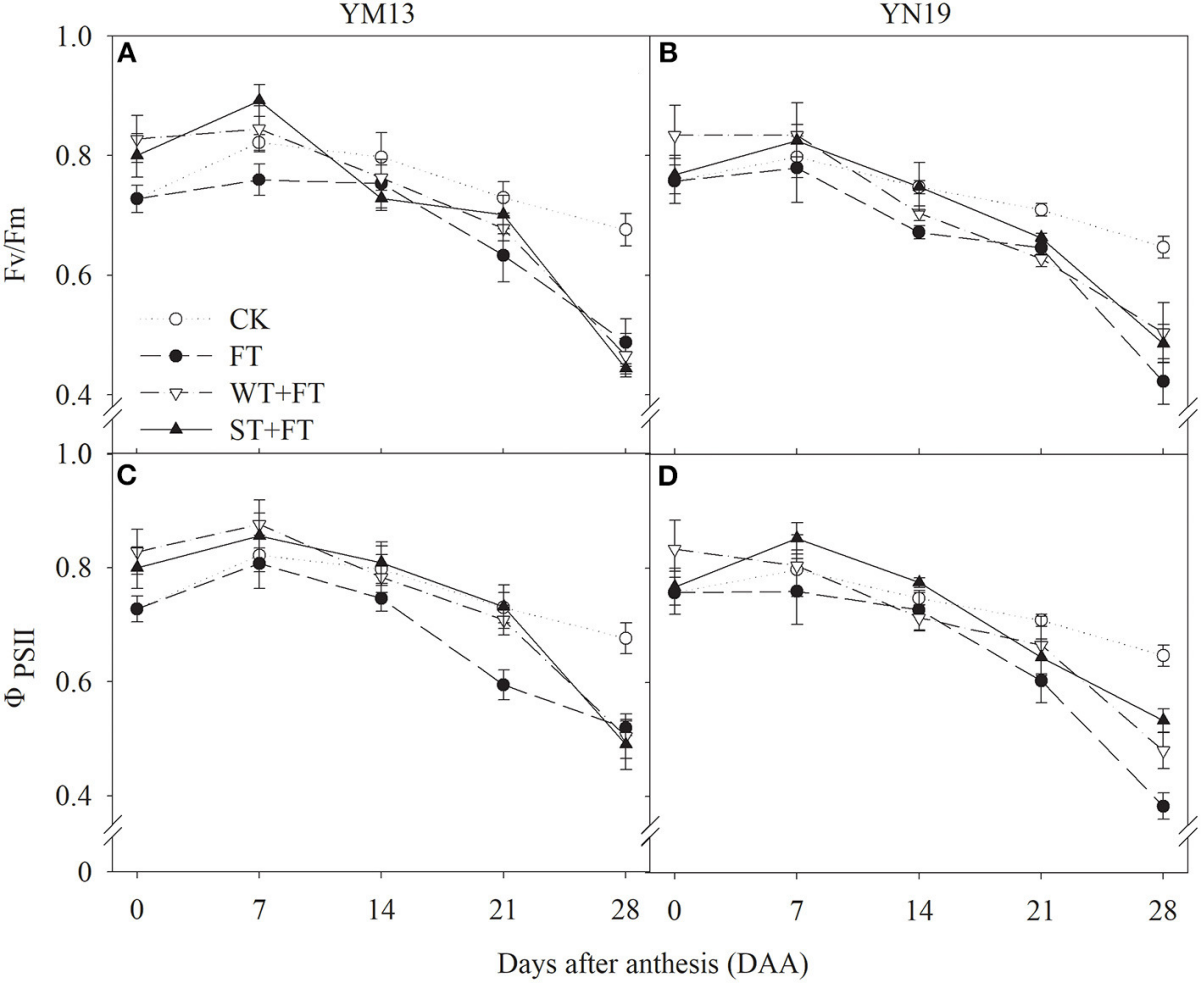
Effect of a night warming treatment in winter or spring on maximum efficiency of photosystem II (PS II) photochemistry under dark-adapted (Fv/Fm) **(A,B)** and the effective quantum yield of PS II photochemistry (ΦPSII) **(C,D)** in wheat flag leaves later treated with night warming during the grain-filling period.

Figures 4A,B shows that the F_v_/F_m_ of the flag leaves was higher under WT + FT and ST + FT than under the control treatment from 0 to 7 DAA, lower under WT + FT and ST + FT than under the control treatment from 14 to 28 DAA (this difference increased over time during the grain-filling period), and always higher under WT + FT and ST + FT than under FT. There was a significant difference in the flag leaf F_v_/F_m_ between warming treatments at 0–28 DAA (*p* < 0.01; Table 3). Figures 4C,D shows that the Φ_PSII_ of flag leaves was higher under WT + FT and ST + FT than under the control treatment from 0 to 7 DAA and higher under WT + FT and ST + FT than under FT from 0 to 21 DAA.

### Soluble Protein and Rubisco Content

**Figure 5:**
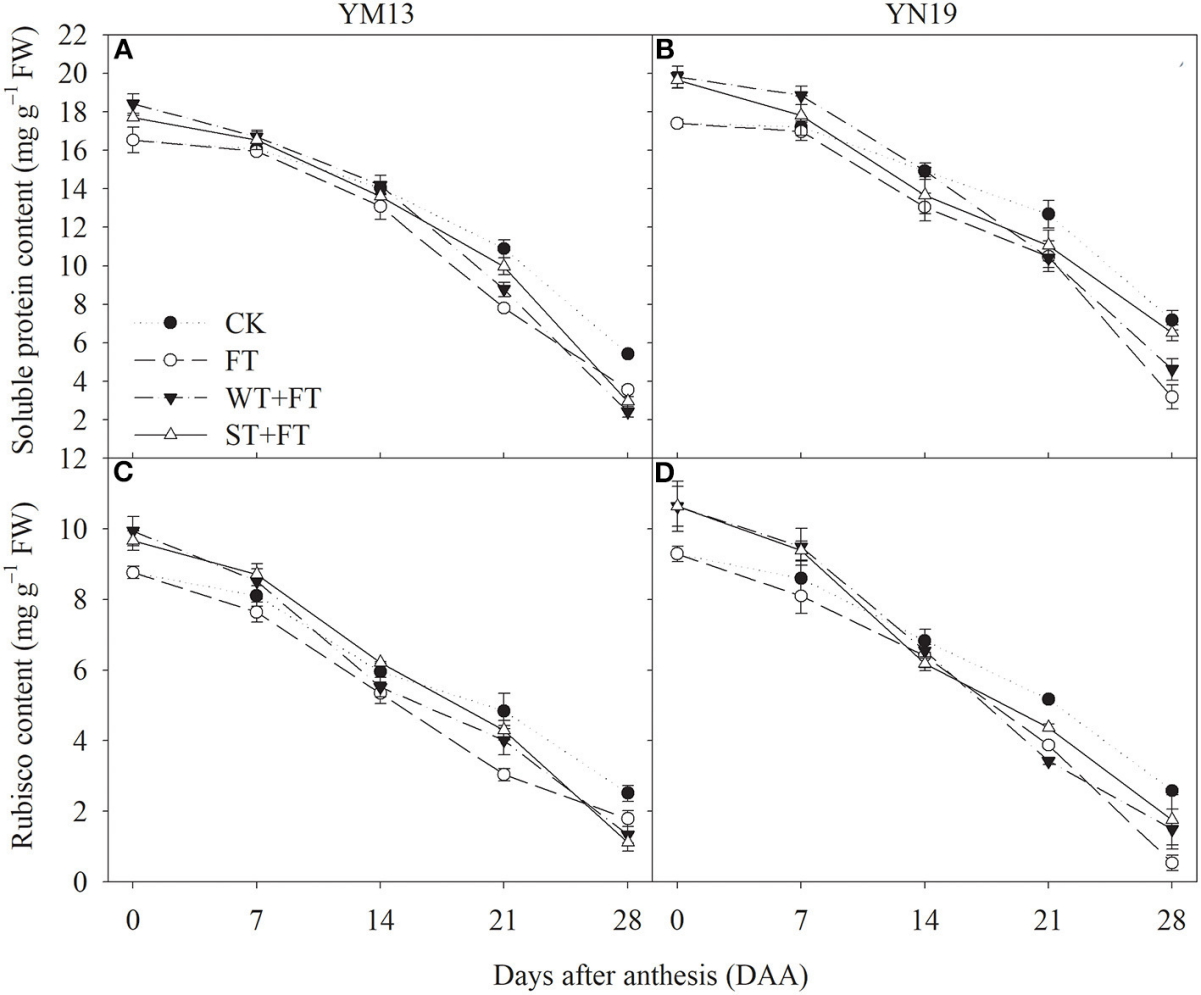
Impact of night warming treatment in winter or spring on the soluble protein content **(A,B)** and Rubisco content **(C,D)** in wheat flag leaves later treated with night warming during the grain-filling period.

Figures 5A,B shows that FT reduced the soluble protein content of flag leaves after anthesis, and this reduction gradually increased over time in the grain-filling period. The soluble protein contents of flag leaves were higher under WT + FT and ST + FT than under the control treatment from 0 to 7 DAA and lower under WT + FT and ST + FT than under the control treatment from 21 to 28 DAA. Night-warming priming alleviated the FT-induced reduction in soluble protein content in flag leaves during the grain-filling period. Figures 5C,D shows that the differences in the post-anthesis Rubisco contents between treatments followed a trend similar to that of the soluble protein contents, suggesting that a primed plant resulted in stronger photosynthetic carbon assimilation in flag leaves in the early grain-filling period and alleviated the FT-induced damage to the photosynthetic system during the middle to late grain-filling period.

### Degree of Oxidative Metabolism

**Figure 6:**
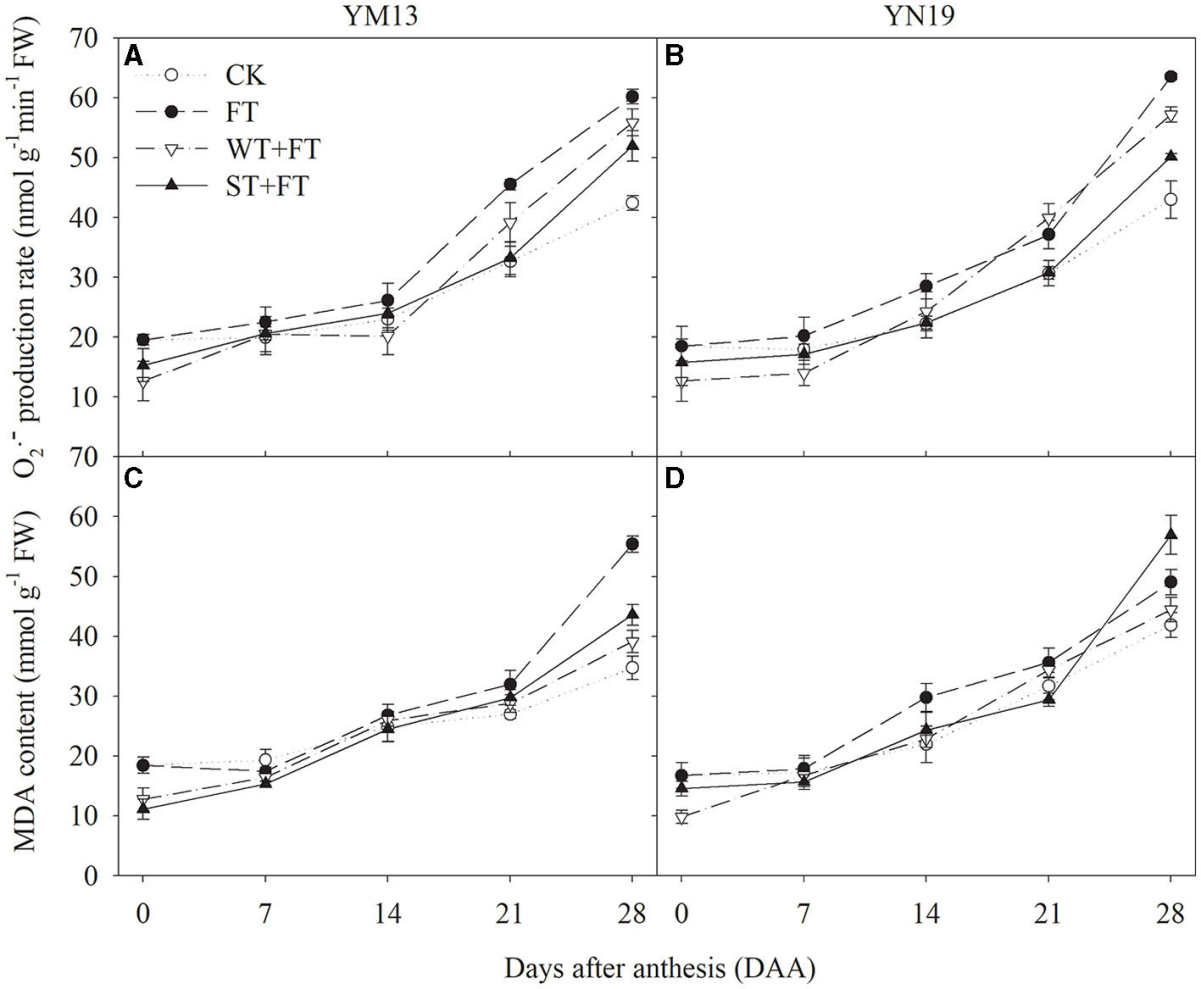
Impact of a night warming treatment in winter or spring on the rate of superoxide anion generation (${\text{O}}_{2}^{•-}$) **(A,B)** and the malondialdehyde level (MAD) **(C,D)** in wheat flag leaves later treated with night warming during the grain-filling period.

Figures 6A,B shows that FT increased the production rate of ${\text{O}}_{2}^{-}$ in the post-anthesis flag leaves, indicating that FT accelerated the senescence of flag leaves. The production rates of ${\text{O}}_{2}^{-}$ in flag leaves were lower under WT + FT and ST + FT than under the control treatment during the anthesis period. This was accompanied by increasingly higher rates of senescence after anthesis, which was significantly higher under WT + FT and ST + FT than under the control treatment at 28 DAA, and always lower under WT + FT and ST + FT than FT at 0–14 DAA. Figures 6C,D shows that the MDA concentrations in flag leaves under WT + FT and ST + FT were lower than those under the control treatment during the anthesis period, and they were also lower under WT + FT and ST + FT than under FT at 0–21 DAA. There was a significant difference in the flag leaf production rate of ${\text{O}}_{2}^{-}$ and MDA content between warming treatments at 0, 21–28 DAA (*p* < 0.01; Table 3). These results indicate that night-warming priming alleviated the ROS-induced damage caused by FT.

### Membrane Phospholipid Metabolism

**Figure 7:**
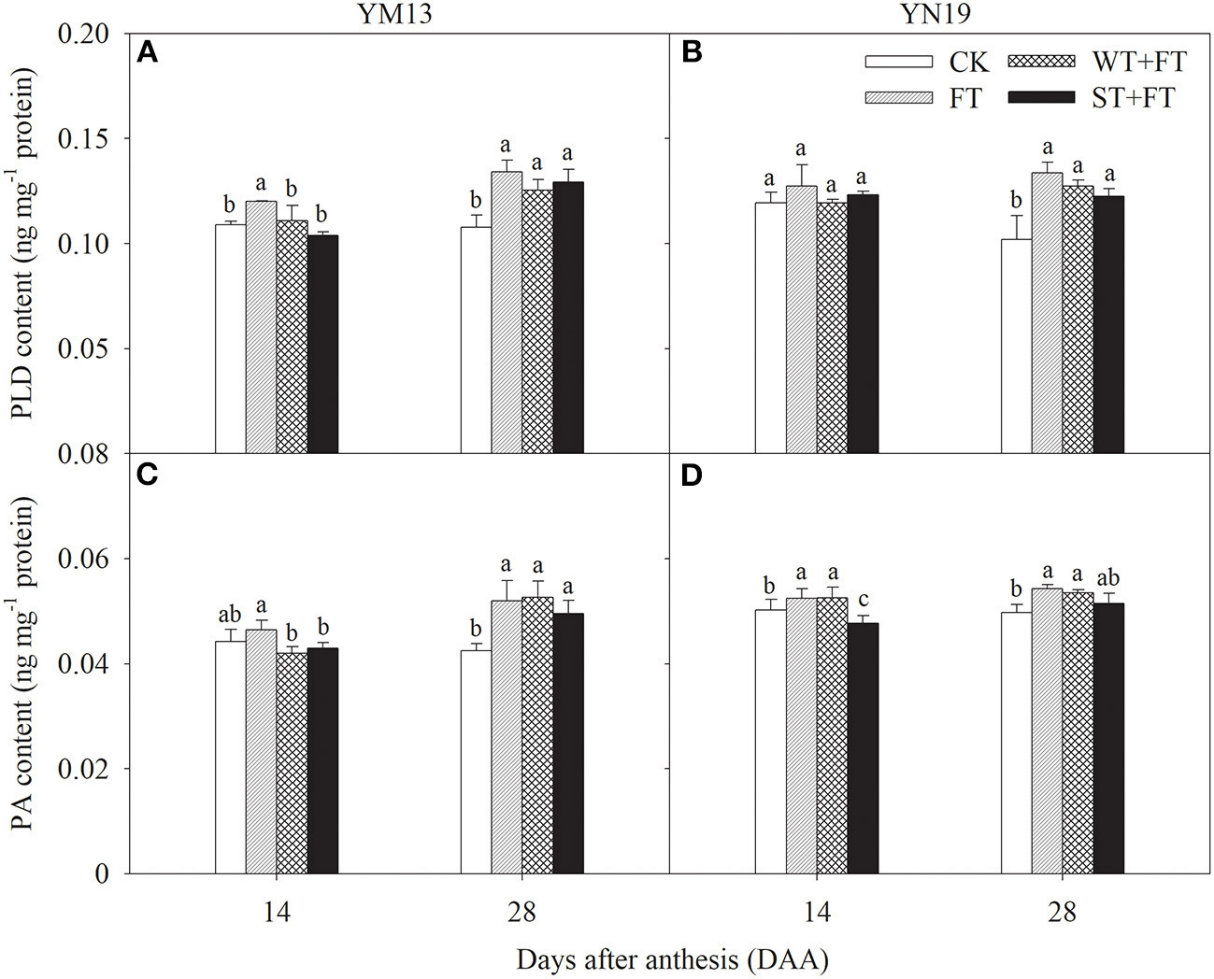
Impact of a night warming treatment in winter or spring on the phospholipase D (PLD) **(A,B)** and phosphatidic acid (PA) contents **(C,D)** in wheat plants later treated with night warming during the grain-filling period. Different lowercase letters indicate significant differences between treatments (*p* < 0.05) hereinafter.

Figures 7A,B shows that the PLD in flag leaves at 14 DAA was higher under FT than under the control treatment, but not significantly different among WT + FT, ST + FT, and the control. The PLD in flag leaves at 28 DAA was significantly higher under all the warming treatments than under the control treatment, and FT had the highest PLD. Figures 7C,D shows that, in YM13, the PA in flag leaves was significantly lower under WT + FT and ST + FT than FT at 14 DAA, and significantly higher under the three warming treatments than under the control treatment at 28 DAA. In YN19, the PA contents in flag leaves were significantly lower under ST + FT than under the control and FT treatment at 14 DAA, were significantly higher under FT and WT + FT than under the control treatment at 28 DAA, and were not significantly different between ST + FT and the control treatment at 28 DAA.

**Figure 8:**
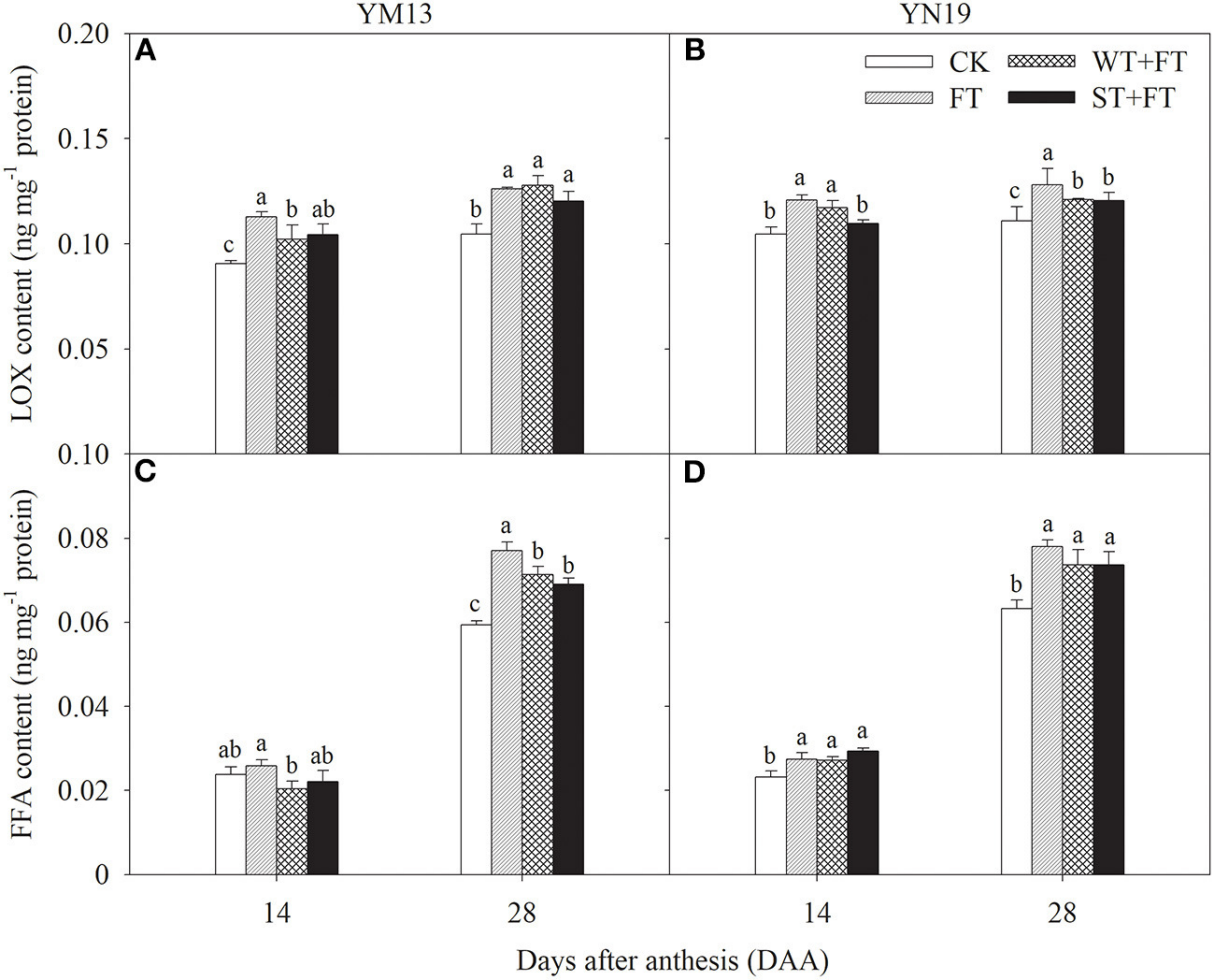
Impact of a night warming treatment in winter or spring on the lipoxygenase (LOX) **(A,B)** and free fatty acid concentrations (FFA) **(C,D)** in flag leaves of wheat plants later treated with night warming during the grain-filling period. Different lowercase letters indicate significant differences between treatments (*P* < 0.05) hereinafter.

Figures 8A,B shows that the LOX in flag leaves at 14 and 28 DAA was higher under the three warming treatments than under the control treatment, which was the highest under FT. Figures 8C,D shows that the FFA in YM13 and YN19 at 28 DAA was significantly higher under the three warming treatments than under the control treatment, following the ranking FT > WT + FT > ST + FT > CK. Thus, FT promoted the degradation of membrane phospholipids by LOX in flag leaves and accelerated the senescence of flag leaves. Night-warming priming alleviated the post-florescence degradation of membrane phospholipids in the flag leaves of wheat plants treated with FT.

### Activities of Antioxidant Enzymes

**Figure 9:**
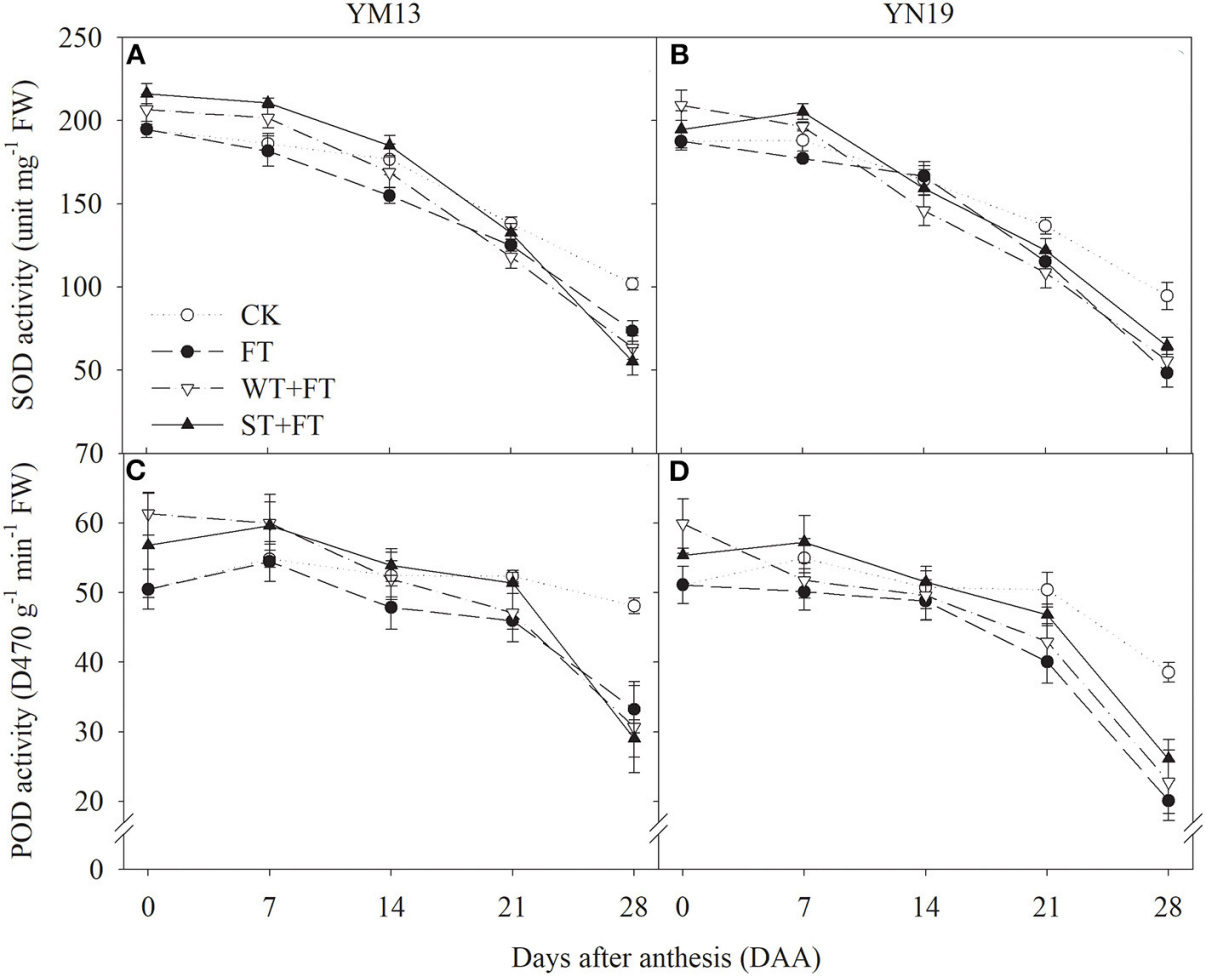
Impact of a night warming treatment in winter or spring on the superoxide dismutase (SOD) **(A,B)** and antioxidant enzyme activities **(C,D)** in the flag leaves of wheat plants later treated with night warming during the grain-filling period.

Figures 9A,B shows that the SOD activity in flag leaves of YM13 and YN19 was higher under WT + FT and ST + FT from 0 to 7 DAA and lower from 21 to 28 DAA than under the control treatment. In particular, WT + FT and ST + FT increased the post-anthesis SOD activity in flag leaves than that under FT. Figures 9C,D shows that FT gradually decreased post-anthesis POD activity during the grain-filling period, while the POD activity in flag leaves of wheat plants treated with WT + FT and ST + FT remained high during the early grain-filling period but gradually decreased and became lower than under the control treatment in the late grain-filling period, but still higher than that under FT from 0 to 21 DAA. There was a significant difference in the flag leaf POD activity between warming treatments at 0–28 DAA (*p* < 0.01; Table 3).

## Discussion

The yields of both wheat cultivars were significantly lower under the three warming treatments than under the control treatment, with the treatments following descending order of CK > WT + FT > ST + FT > FT. In other words, FT resulted in the greatest reduction in wheat grain yield, while WT and ST alleviated the adverse effect of FT on grain yield mainly by increasing the number of grains per panicle and 1,000-grain weight. Heat stress can a?ect the yield components by shortening the grain filling duration due to an accelerated rate of development, accelerated leaf senescence, and the inhibition of photosynthesis and carbohydrate synthesis (Asseng et al., 2011). Pre-stress treatments during vegetative stages (priming) have been shown to increase stress tolerance in plants and alleviate abiotic stress in grasses (Wang et al., 2017; Thayna et al., 2018). Our previous study showed that, compared with the damage observed in non-primed plants, heat priming during the stem-elongation stage and booting significantly prevented the grain-yield damage caused by heat stress during grain-filling (Fan et al., 2018). Tian et al. (2012) indicated that an increased night temperature can increase cell metabolism, the nighttime respiration rate of leaves, and the growth rate of leaves. Under night-warming conditions, the number of leaf cells and the plant metabolism become more vigorous. The above evidence suggests that night-warming priming at the vegetative stage proved to contribute to grain yield sustainability by enhancing the ability of plants to mitigate the effects of warming during the grain-filling stage.

Photosynthesis is the most sensitive physiological process to high temperatures. Depressed photosynthesis under heat stress often negatively affects the growth and grain yield of wheat. Many studies have demonstrated that most plant species can adapt to alterations in growth temperature by adjusting their photosynthetic system to optimize performance in a new temperature environment (Martinez et al., 2014; Liu et al., 2016). In the present study, the flag leaves of primed plants maintained a high *Pn* and chlorophyll content in the early grain-filling period, and their photosynthetic capacity gradually became lower than the capacity of the control group but still higher than that under FT. Lobell et al. (2012) found that a high temperature in the late growth period decreases the photosynthetic capacity of wheat leaves, exacerbates membrane lipid peroxidation, accelerates plant senescence, and reduces wheat yield. Li et al. (2011) showed that the pre-anthesis waterlogging of wheat plants improved their resistance to post-anthesis waterlogging. Liu et al. (2016) reported that post-anthesis heat stress and its combination with drought significantly decreased *Pn*, and the primed plants showed a significantly higher *Pn* than the non-primed plants under post-anthesis drought and heat stress, suggesting that drought priming enhanced the capacity of the plants to protect photosynthetic activity from exposure to a later stress event. These findings suggest that warming and stresses in an early stage can induce the partial memory of stress-response signals in plants through self-regulation, thereby improving their stress tolerance. The chlorophyll fluorescence parameters of plants change significantly under stress, such as high temperature, and are closely related to the heat resistance of plants (Allakhverdiev et al., 2008). In this study, FT reduced the chlorophyll fluorescence parameters of flag leaves, making them unconducive to the electron transfer needed for photosynthesis. The night-warming priming treatment reduced the negative impact of post-anthesis warming on the chlorophyll fluorescence parameters of wheat flag leaves, thereby maintaining the electron transfer in photosynthesis and the high light use efficiency in flag leaves of wheat plants. This finding indicates that, compared with the non-primed treatment, night-warming priming at the vegetative stage enhanced the photosynthetic capacity and stress tolerance in the flag leaves of winter wheat, which benefited the grain-filling process.

The enzyme Rubisco catalyzes the assimilation of CO_2_ by the carboxylation of ribulose diphosphate carboxylase (RuBP) in the Calvin cycle. Rubisco accounts for 50% of the leaf's total soluble protein content and is, therefore, the most obvious target for improving the photosynthetic capacity of crops (Galmés et al., 2014). In this study, the soluble protein and Rubisco contents in post-anthesis flag leaves of the primed plants were higher than those in post-anthesis flag leaves treated with post-anthesis warming. This means that night-warming priming at the vegetative stage could improve flag leaf's photosynthetic carbon assimilation ability and be conducive to the accumulation of photosynthetic products. The response mechanism that resisted the high-temperature stress could have been generated in wheat plants after the night-warming adaptation during winter and spring, which could have prompted a stress-induced tolerance of the wheat plants to post-anthesis warming. The soluble protein content in plant leaves reflected the nitrogen content, and the nitrogen content in the leaves had a positive relation with photosynthetic capacity. Most of the nitrogen in plants is stored in the enzymes that participated in the photosynthesis, especially in Rubisco, which is a major source of nitrogen recycle (Masclaux-Daubresse et al., 2008). The above evidence suggests that the Rubisco and soluble protein contents were relatively high at the post-anthesis stage in the primed plants, which was conducive to increasing photosynthetic capacity and grain yield.

Leaf senescence is associated with increased cellular ROS and increased membrane lipid peroxidation (Lu et al., 2001). Pre- and post-anthesis heat stress has already been shown to reduce photosynthetic rates in wheat through oxidative damage, which accelerates leaf senescence (Wang et al., 2011, 2012). Under heat-stress conditions, higher photosystem efficiency also prevents ROS generation and assists rapid and complete photosystem recovery at normal temperatures (Li et al., 2014). The results of this study showed that, under pre-anthesis night warming, the ${\text{O}}_{2}^{-}$ production rate and MDA content in post-anthesis leaves were lower under WT + FT and ST + FT than under FT, indicating that night-warming priming reduced membrane lipid peroxidation in the flag leaves of wheat plants treated with post-anthesis warming. In this study, the behavior of phospholipid metabolism in flag leaves indicated that FT aggravated membrane lipid peroxidation in flag leaves and increased the PLD and LOX contents and PA and FFA production. This result indicates that night-warming priming effectively alleviated the FT-induced degradation of membrane phospholipids in wheat flag leaves, thus alleviating senescence. Wang et al. (2011) reported that pre-anthesis heat priming increases grain yield against subsequent high temperatures during the grain-filling stage, and their finding was attributed to the improved photosynthetic and antioxidative activity in the acclimated plants. Furthermore, SOD and POD, as endogenous protective enzymes, can remove toxic substances, such as ROS, produced during metabolic processes, thereby reducing membrane lipid peroxidation, maintaining normal cellular metabolism, and delaying leaf senescence (Noodén et al., 1997; Zhang et al., 2015). Jing et al. (2020) found that warming in late winter and early spring significantly increased the SOD and POD activities of flag leaves and effectively alleviated their senescence. The results of our study indicate that FT impairs the maintenance of the activity of the antioxidative protection system in post-anthesis flag leaves and that night-warming priming can alleviate the inhibitory effects of FT on the activities of antioxidant enzymes, thus alleviating peroxidative stress. This result indicated that night-warming priming at the vegetative stage increased the antioxidant capacity of flag leaves treated with FT, helped maintain higher cell membrane thermo-stability (an essential trait related to heat stress tolerance) and the balance between the generation and elimination of ROS, and thereby improved the high-temperature tolerance of post-anthesis wheat plants.

## Conclusions

In summary, the wheat plants treated with winter and spring night warming maintained a high photosynthetic capacity and strong ROS scavenging ability in flag leaves, thus reducing membrane lipid peroxidation and improving plant tolerance, which then contributed to yield formation. Night-warming priming at the vegetative stage proved to be a valuable strategy for triggering plants to initialize an efficient tolerance mechanism, which, in turn, permitted the plants to tolerate post-anthesis warming conditions. Thus, knowledge of the mechanisms underlying temperature adaptation and acclimation in wheat cultivars offers another perspective for understanding how crop performance can be improved under changing climate conditions.

## Data Availability Statement

The original contributions presented in the study are included in the article/supplementary material, further inquiries can be directed to the corresponding authors.

## Author Contributions

YF, ZL, ZH, and TD designed the experiment. YF and ZL conducted the study, collected and analyzed the data, and prepared the draft. TG, YL, and WY helped in sampling and measuring physiological parameters. WZ and SM helped draft the manuscript and interpret the results. All authors contributed to the article and approved the submitted version.

## Funding

This work was supported by the National Natural Science Foundation of China (U19A2021), the Project of Natural Science Foundation of Anhui Province (2008085qc118), and the Major Science and Technology Special Project of Anhui Province (S202003a06020035). We also acknowledge the support from the Jiangsu Collaborative Innovation Center for Modern Crop Production (JCIC-MCP).

## Conflict of Interest

The authors declare that the research was conducted in the absence of any commercial or financial relationships that could be construed as a potential conflict of interest.

## Publisher's Note

All claims expressed in this article are solely those of the authors and do not necessarily represent those of their affiliated organizations, or those of the publisher, the editors and the reviewers. Any product that may be evaluated in this article, or claim that may be made by its manufacturer, is not guaranteed or endorsed by the publisher.
